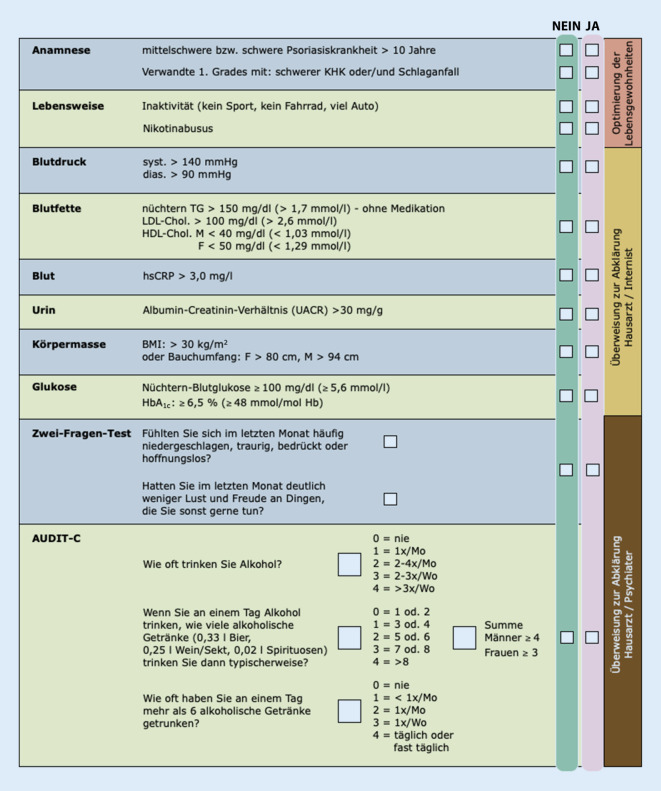# Handlungsempfehlungen zur individuellen Risikoermittlung von Komorbidität bei erwachsenen Patienten mit Psoriasis

**DOI:** 10.1007/s00105-023-05116-7

**Published:** 2023-02-22

**Authors:** Johannes Wohlrab, Andreas Körber, Georg Adler, Matthias Blüher, Andreas Zirlik, Sascha Gerdes

**Affiliations:** 1 Universitätsklinik für Dermatologie und Venerologie, Martin-Luther-Universität Halle-Wittenberg, Ernst-Grube-Str. 40, 06120 Halle (Saale), Deutschland; 2 Hautärzte Rue143, Essen, Deutschland; 3 Institut für Studien zur Psychischen Gesundheit (ISPG), Mannheim, Deutschland; 4 Helmholtz Institut für Metabolismus‑, Adipositas- und Gefäßforschung (HI-MAG), Universität Leipzig, Leipzig, Deutschland; 5 Universitäres Herzzentrum Graz, Klinische Abteilung für Kardiologie der Klinik für Innere Medizin, Medizinische Universität Graz, Graz, Österreich; 6 Klinik für Dermatologie, Venerologie und Allergologie (Psoriasis-Zentrum), Universitätsklinikum Schleswig-Holstein, Campus Kiel, Kiel, Deutschland

**Keywords:** Psoriasis, Komorbidität, Metabolisches Syndrom, Kardiovaskuläres Risiko, Psychische Erkrankungen, Psoriasis, Comorbidity, Metabolic syndrome, Cardiovascular risk, Mental illness

## Abstract

Es ist seit Langem bekannt, dass chronisch entzündliche Systemerkrankungen wie die Psoriasis ein hohes Risiko für die Entwicklung von Komorbidität bieten. Im klinischen Alltag ist es deshalb von besonderer Bedeutung, Patient:innen zu identifizieren, die ein individuell erhöhtes Risikoprofil bieten. Bei Menschen mit Psoriasis konnten in epidemiologischen Studien in Abhängigkeit von Krankheitsdauer und -schwere die Komorbiditätsmuster „metabolisches Syndrom“, „kardiovaskuläre Komorbidität“ und „psychische Erkrankungen“ als besonders relevant identifiziert werden. In der alltäglichen Versorgung von Menschen mit Psoriasis in der dermatologischen Praxis haben sich der Einsatz einer interdisziplinär inhaltlich abgestimmten Checkliste für die Risikoanalyse und die Bahnung einer professionellen Anschlussversorgung bewährt. Auf der Basis einer existierenden Checkliste wurden die Inhalte von einer interdisziplinären Expertengruppe kritisch bewertet, und eine leitlinienorientierte Aktualisierung wurde vorgenommen. Der nun vorgelegte Analysebogen stellt nach Auffassung der Autoren ein praktikables, sachbezogen fokussiertes und inhaltlich aktualisiertes Werkzeug für die Risikoermittlung von Komorbidität bei Patient:innen mit mittelschwerer bis schwerer Psoriasis dar.

## Abstract (en)

It has long been known that chronic inflammatory systemic diseases, such as psoriasis, pose a high risk of developing comorbidities. In everyday clinical practice, it is therefore of particular importance to identify patients who have an individually increased risk profile. In patients with psoriasis, the comorbidity patterns “metabolic syndrome”, “cardiovascular comorbidity” and “mental illness” were identified as particularly relevant in epidemiological studies depending on the duration and severity of the disease. In the everyday care of patients with psoriasis in dermatological practice, the use of an interdisciplinary checklist for risk analysis and the initiation of professional follow-up care has proven valuable. On the basis of an existing checklist, the contents were critically evaluated by an interdisciplinary group of experts and a guideline-oriented update was prepared. In the opinion of the authors, the new analysis sheet represents a practicable, factually focused and updated tool for comorbidity risk assessment in patients with moderate and severe psoriasis.

Psoriasis wird heute als eine genetisch disponierte, autoimmunologisch und autoinflammatorisch vermittelte, chronisch entzündliche Systemerkrankung (Psoriasiskrankheit) verstanden, die phänotypisch insbesondere die Haut (Psoriasis vulgaris), Gelenke, gelenknahen Knochen und Enthesen (Psoriasisarthritis) betrifft [40, 66]. Im Zentrum der Immunpathogenese wird dabei die Aktivierung von naiven T‑Zellen durch dendritische Zellen gesehen, die zytokinvermittelt (z. B. IL-23, IL‑6, TGF-β) in pathologische und regulatorische Th17-Zellen differenzieren. Diese wiederum exprimieren Botenstoffe, insbesondere Subtypen von IL-17, TNF-α und IL-22, die über Rezeptoren auf Effektorzellen in verschiedenen Organen pathologische Reaktionsmuster induzieren. Darüber hinaus werden über „pathogen-associated molecular patterns“ (PAMPS) und „damage-associated molecular patterns“ (DAMPS) Toll-like(TLR)- und Nod-like-Rezeptoren (NLR) aktiviert, die als Teil des NLRP3-Inflammasoms vorwiegend über Pro-Caspase 1 zur Expression von Zytokinen der IL-1-Familie führen [43, 44, 66]. Dadurch wird die Immunpathogenese durch autoimmunologische und autoinflammatorische Phänomene individuell variabel bedingt. Beide pathogenetischen Kaskaden sind Teil eines systemischen Entzündungsgeschehens, welches in Abhängigkeit von individuellen genetischen und epigenetischen Faktoren sowie der Schwere und Dauer der Erkrankung zur Komorbidität beitragen [21, 38, 54, 61]. Gleichzeitig lassen sich aus diesen Zusammenhängen Strategien für präventive bzw. therapeutische Ansätze sowohl für die Grunderkrankung selbst als auch für die komorbiden Symptome ableiten.

Es werden verschiedene Symptomkomplexe von Komorbidität unterschieden, deren klinische Relevanz sehr unterschiedlich sein kann und die bereits im Kindesalter beobachtet werden können [51]. Aufgrund der Häufigkeit und der sich daraus ableitenden klinischen Bedeutung sind aus dermatologischer Perspektive insbesondere die Symptomkomplexe metabolisches Syndrom, kardiovaskuläre Komorbidität und psychische Erkrankungen von hoher Relevanz [28]. Um die Ermittlung des individuellen Risikos für Komorbidität bei Patient:innen mit einer mittelschweren bis schweren Psoriasis praktikabel zu gestalten, wurden für den klinischen Alltag Handlungsempfehlungen erarbeitet, die in Form von Checklisten und Fragebögen eine Abschätzung ermöglichen [32, 52, 70]. Die Anwendung von derartigen Checklisten im Alltag hat sich aus Sicht der Autoren besonders bewährt. Dieses Vorgehen bedarf aber einer stetigen inhaltlichen Überprüfung, um sowohl die aktuelle Evidenz zu den jeweiligen Komorbiditätsmustern interdisziplinär abzubilden, als auch die fachspezifischen Therapien für die Versorgung der Patient:innen leitlinienkonform zu bahnen [17, 62]. Zudem verdichten sich die Hinweise, dass eine konsequente Langzeittherapie zur Reduktion der Aktivität der Psoriasis das Risiko für Komorbidität relevant reduziert [10, 53].

Auf der Grundlage einer bereits etablierten Handlungsempfehlung möchten die Autoren im Rahmen eines interdisziplinären Diskurses eine kritische Analyse sowie Aktualisierung einer etablierten Checkliste vornehmen und damit der aktuell vorliegenden Evidenz Rechnung tragen.

## Metabolisches Syndrom

Auch wenn die Definition eines metabolischen Syndroms Unschärfen aufweist, so wird heute darunter ein komorbides und sich gegenseitig akzelerierendes Auftreten von Typ-2-Diabetes, Übergewicht, Hypercholesterinämie und arterieller Hypertonie verstanden [4, 49, 57, 63]. In engem pathogenetischen Zusammenhang bzw. gehäufter Koexistenz werden zudem eine nichtalkoholische Fettleber (NAFLD), eine Nephropathie (Albumin-Kreatinin-Verhältnis im Urin [UACR] > 30 mg/g), eine obstruktive Schlafapnoe (OSA) bzw. ein polyzystisches Ovarialsyndrom (PCOS) beobachtet [24, 57]. Patient:innen mit einer Psoriasis haben in Abhängigkeit von Erkrankungsschwere und -dauer ein erhöhtes Risiko, ein metabolisches Syndrom als Komorbidität zu entwickeln [6, 47]. Umgekehrt stellt das metabolische Syndrom einen Risikofaktor für das Auftreten einer Psoriasis dar [29, 37]. Als Diagnosekriterien für ein metabolisches Syndrom gelten der Bauchumfang bei Männern von mehr als 94 cm und bei Frauen von mehr als 80 cm sowie das Vorliegen von mindestens 2 weiteren der folgenden Störungen: arterielle Hypertonie (systolisch ≥ 130 mm Hg, diastolisch ≥ 85 mm Hg als Mittelwert einer 24-h-Langzeitblutdruckmessung oder alternativ systolisch ≥ 140 mm Hg, diastolisch ≥ 90 mm Hg als wiederholte Einzelmessung) oder eine bereits behandelte arterielle Hypertonie, Nüchtern-Triglyzeride ≥ 150 mg/dl (≥ 1,7 mmol/l) (ohne Medikation), HDL-Cholesterin < 40 mg/dl (< 1,03 mmol/l) bei Männern und < 50 mg/dl (< 1,29 mmol/l) bei Frauen, erhöhte Nüchtern-Blutglukose ≥ 100 mg/dl (≥ 5,6 mmol/l) bzw. HbA_1c_ ≥ 6,5 % (≥ 48 mmol/mol) oder ein bereits diagnostizierter Typ-2-Diabetes [2, 72]. Daraus ergibt sich für Personen mit einem metabolischen Syndrom ein 2‑ bis 3fach erhöhtes Risiko für ein unerwünschtes kardiovaskuläres Ereignis (MACE) [5, 27, 35]. Grundsätzlich sollte deshalb neben einer Ernährungs- und Lebensstilberatung die Gabe von Antihypertensiva, Statinen sowie von Antidiabetika erwogen und fachärztlich abgeklärt werden.

## Kardiovaskuläre Komorbidität

Die Bedeutung der kardiovaskulären Komorbidität bei Menschen mit Psoriasis ist bereits epidemiologisch gut untersucht, und die Hypothesen für die pathogenetischen Zusammenhänge sind weitgehend durch Evidenz belegt [7, 8, 19, 25, 48]. Bereits bei jugendlichen Patient:innen lassen sich entsprechende Veränderungen nachweisen [34]. Im Zentrum der pathologischen Vorgänge im kardiovaskulären System steht die sich in Abhängigkeit von Erkrankungsschwere und -dauer zunehmend etablierende Atherosklerose, die durch die psoriatische Entzündung wesentlich getrieben wird [23]. Dies konnte sowohl durch einen Anstieg von Biomarkern der Entzündung (hsCRP, VEGF, P‑Selektin) [9, 60, 65] als auch durch bildgebende Verfahren zum Nachweis der vaskulären Entzündung (18F-Fluorodeoxyglukose-Positronenemissions-Computertomographie [FDG-PET-CT]) belegt werden [45]. Der molekulare Zusammenhang wird unter anderem in einer zytokinvermittelten Insulinresistenz und einer sich daraus ableitenden endothelialen Dysfunktion gesehen [6, 31]. Letztere führt durch ein verändertes Insulinsignal an Endothelzellen über eine reduzierte Aktivität der endothelialen Stickstoffmonoxidsynthase (eNOS) und verminderter Expression von Endothelin 1 (ET-1) zu einer Gefäßdilatation und bahnt die proinflammatorischen Milieubedingungen des atherosklerotischen Umbaus [23, 30, 46]. Die sich daraus ableitende chronische Entzündung der Gefäßwände mit Zunahme der Intima- und Mediadicke sowie Ausbildung von Kalzifikationen an den Koronararterien bedingen dann die definierten klinischen Ereignisse wie Herzinfarkt bzw. Schlaganfall [11, 39]. Vor diesem Hintergrund lassen sich aus klinischer Perspektive abhängige und unabhängige Faktoren formulieren, an denen sich das individuelle Risiko eines Patienten abschätzen lässt. Neben familienanamnestischen Angaben zu unerwünschten kardiovaskulären Ereignissen (MACE) und Nikotinabusus ist v. a. das Vorliegen einer arteriellen Hypertonie von Bedeutung [52, 70]. Zudem wird überlappend auch das Vorliegen eines metabolischen Syndroms mit LDL-Hypercholesterinämie, Übergewicht und Typ-2-Diabetes als bedeutsam für das kardiovaskuläre Risiko gesehen [59].

## Psychische Erkrankungen

Die Psoriasis kann eine erhebliche psychosoziale Belastung darstellen. Darüber hinaus fungiert die Haut auch als neuroendokrines Organ, und die Psoriasis kann als chronisch entzündliche Systemerkrankung über Botenstoffe durch metabolische Prozesse Einfluss auf das Nervensystem nehmen, sodass eine funktionelle Verquickung der beiden Organsysteme vorliegt [71]. Neben einer naheliegenden Interaktion zwischen Haut und dem peripheren Nervensystem im Rahmen von Juckreiz oder Parästhesien [33] hat sich auch das Verständnis der Einflussnahme auf das zentrale Nervensystem (ZNS) entwickelt und kann mittlerweile auf verschiedenen Ebenen mit Evidenz belegt werden [13, 26, 41]. Aus klinischer Sicht sind insbesondere Depression, Angststörung und Suizidalität, Suchterkrankung und sozialer Rückzug von Bedeutung [1, 18, 20, 55, 56]. Die Identifizierung des erhöhten individuellen Risikos bei Patient:innen bzw. die Erkennung von Markersymptomen durch die Dermatolog:in ist v. a. von Bedeutung, weil psychopharmakotherapeutische, somatotherapeutische oder psychotherapeutische (z. B. interpersonelle Psychotherapie) Interventionen, insbesondere bei Angststörung und Depression, ein wirksames Instrument zur Behandlung der Komorbidität darstellen können [42]. Neben dem sozioökonomischen Status der Patient:in und den damit verbundenen individuellen Lebensumständen lassen sich weitere, unabhängige Risikofaktoren definieren [14, 64]. Mit Blick auf die Erfassung einer depressiven Störung bietet der „Zwei-Fragen-Test“ mit einer hohen Sensitivität (ca. 96 %) bei mittlerer Spezifität (ca. 57 %) für die schnelle Erfassung eine geeignete Möglichkeit [22, 50, 69]. Darüber hinaus gibt es zur Erfassung von Depression deutlich aussagekräftigere psychometrische Tools, die aber für die zeitsparende Erfassung im vorliegenden Kontext weniger sinnvoll erscheinen [36, 67, 68]. Zur Behandlung einer depressiven Störung hat sich zudem die Gabe von Citalopram in einer Dosierung von 10–20 mg/Tag als nützlich, praktikabel und unproblematisch erwiesen [15]. Für die Identifikation einer Alkoholsucht (Risiko für eine alkoholbezogene Störung) wird der *Alcohol Use Disorders Identification Test – Consumption Items* (AUDIT-C) empfohlen und ist im vorliegenden Zusammenhang geeignet [3, 12, 16]. Um eine aussagefähigere Einschätzung vornehmen bzw. andere Suchterkrankungen und -aspekte erfassen zu können, sind weiterführende Verfahren möglich [58]. Insgesamt lässt sich feststellen, dass die ausgewählten Tests (Zwei-Fragen-Test und AUDIT-C) für die rasche und zielorientierte Ermittlung von Risikopatient:innen ein praktikables, validiertes und aussagefähiges Verfahren darstellen.

## Hinweise zum Einsatz der Checkliste

Die Checkliste ist erarbeitet worden, um eine praktikable und fokussierte Ermittlung des Risikos auf Komorbidität einer individuellen Patient:in im klinischen Alltag abschätzen zu können (Abb. 1). Die gewählten Parameter orientieren sich an den für die Diagnostik der einzelnen Komorbiditätsmuster etablierten Verfahren und den aktuellen Leitlinien bezogen auf die jeweilige Komorbidität. Sie sind aber explizit nicht für eine fundierte Diagnosestellung ausreichend, sondern sollen der Dermatolog:in die Identifizierung von Patient:innen mit besonderer Risikokonstellation ermöglichen, um eine fachärztlich spezialisierte Betreuung im Rahmen der interdisziplinären Versorgung der Psoriasiskrankheit bahnen zu können. Es wird empfohlen, die Checkliste vor Therapiebeginn und anschließend jährlich anzuwenden. Bei entsprechender Risikokonstellation ist die ausgefüllte Checkliste gut geeignet, der Hausärzt:in bzw. der weiterbehandelnden Fachärzt:in eine Begründung für die Überweisung an die Hand zu geben. Ziel ist es, eine möglichst optimierte Versorgung von Patient:innen mit mittelschwerer bis schwerer Psoriasis um den Aspekt der Identifizierung und professionellen Behandlung von Komorbidität zu erweitern. Die Lotsenfunktion der Dermatolog:in ist in diesem Zusammenhang von großer Bedeutung. Die vorliegende Checkliste soll für die interdisziplinäre Schnittstelle ein arbeitstechnisches Werkzeug mit geringem Aufwand und fundierter Aussage für die tägliche Praxis für Dermatolog:innen bereitstellen.

**Figure 1:**